# Multipath Detection and Mitigation of Random Noise Signals Propagated through Naturally Lossy Dispersive Media for Radar Applications

**DOI:** 10.3390/s23239447

**Published:** 2023-11-27

**Authors:** Ana Vazquez Alejos, Muhammad Dawood

**Affiliations:** 1 atlanTTic, Escola de Enxeñaría de Telecomunicación, Universidade de Vigo, 36310 Vigo, Spain; 2 Klipsch School of ECE, New Mexico State University, Thomas & Brown Hall, Las Cruces, NM 88003-8001, USA; muhammad@nmsu.edu

**Keywords:** binary code, correlation function, dispersive propagation, frequency domain, mitigation, multipath, random noise, sidelobe

## Abstract

This paper describes a methodological analysis of the Brillouin precursor formation to understand the impairments undergone by like-noise and random noise waveforms propagating through naturally dispersive media commonly found in radar applications. By means of a frequency-domain methodology based on considering the frequency response of the medium under study, the effect of these dispersive media on the evolution of an input signal can be seen as frequency filtering. The simulations were performed at a center frequency of 1.5 GHz and for a signal bandwidth of 3 GHz. Four random noise signals were considered: Barker codes, PRBS codes, Frank codes, Costas codes and additive white Gaussian noise. The experienced impairments were assessed in terms of cross-correlation function (CCF) degradation. The differences in the behavior of each type of phase and frequency coded signal to face the dispersive propagation have been demonstrated in terms of parameters used for information retrieval: peak amplitude decay, CCF secondary sidelobe level and multipath detectability. Finally, a frequency filtering approach is proposed to mitigate the impairments due to dispersive propagation under multipath conditions.

## 1. Introduction

In radar, the use of like-noise featured signals has been commonly extended due to the derived benefits, such as the low probability of detection, the spectral efficiency and the robustness to interferers. Among others, the main like-noise signals are the Barker, PRBS, Frank and Costas codes, as well as random noise [1,2,3,4]. The latter is usually simulated considering additive white Gaussian noise (WGN) and besides all the aforementioned benefits, the WGN waveform can additionally provide a higher level of security, since if the original transmitted signal is unknown, an unauthorized receiver cannot achieve the information retrieval. Then, the immunity to interferers is especially large for a random noise signal [5,6].

Different scenarios of the radar applications involve the transmission of the noise signals through media with dielectric properties showing frequency-dependent behavior [7,8,9,10,11,12]. The impairments introduced in the transmitted signal due to frequency dispersion can lead the receiver structures to work under optimal performance, thus increasing the difficulty to accomplish accurate retrieval of the carried information—that may consist of bit streaming, amplitude or phase levels, or target presence detection—or just to restore the received signal. 

It has been demonstrated that the propagation of any kind of signal through any dispersive media can arise from the formation of signals known as Brillouin precursors [13,14,15,16,17,18,19,20,21,22,23,24,25,26,27,28,29,30,31,32,33]. This phenomenon depends on the dielectric properties of the underlying medium as well as other parameters or settings; for example, the input signal type and its configuration, the involved transmitted and/or received bandwidth are among the main parameters that additionally determine the formation of the Brillouin precursor [26].

The many references found in the literature [13,14,15,16,17,18,19,20,21,22,23,24,25,26,27,28,29,30,31,32,33] agree in distinguishing the most relevant property of the Brillouin forerunner as the non-exponentially decay of the peak amplitude through the dispersive medium. This feature implies a travelling signal that can ensure that the forerunner formation could reach a larger propagation distance inside the medium of interest. This characteristic may be of great importance for radar applications involving naturally lossy dispersive media, such as water, foliage or soil.

However, despite the obvious advantages, we cannot ignore a set of counterpoints exclusively linked to the Brillouin precursor formation. The most important disadvantage is the downshift undergone by the carrier component. This downshift is inversely related to the broadening experienced by a travelling pulse in its time width. For the case of a sequence of pulses [23] at a given propagation distance, the broadening can lead to a destructive merge of the information which would make the information retrieval impossible and totally erroneous.

If the demodulation stage includes a matched filter or a correlator, the broadening and amplitude level distortion undergone by the transmitted pulses will introduce uncertainty or noise, leading to a larger degradation of the cross-correlation function (CCF), and consequently masking the return echoes detection. The codes analyzed here have mostly been designed with the aim to reduce the sidelobe level issue that usually affects to CCF-based receivers or signal processing [34]. In this paper we show that one of the most important impairments observed in the CCF of a signal affected by frequency dispersion is the presence of sidelobes with a distorted shape and larger amplitude level, which can completely mask weak reflections that provide valuable information on target presence. 

Besides the increase of the secondary sidelobe levels (SSL), another important effect consisted of the main CCF peak width broadening. This impairment becomes critical for the multipath detection capability, given that this latter feature is a function of the pulse/bit period, which could result in a merge with the main CCF peak, thus leading to erroneous target estimation. The study presented here could be extended to Golay codes, which lack secondary lobes by definition [35,36]. Despite this, these codes would not be exempt from experiencing the time broadening of the main peak of the CCF.

Whilst the Barker, PRBS and Frank cases consist of binary phase coded sequences, the Costas case is a frequency coded signal with propagation features slightly different due to the lack of phase transitions between adjacent bits [23]. Costas sequences, also named Costas arrays, find their main applications in radar and sonar via the Doppler effect [37,38,39]. It is considered that using a Costas array pattern increases the performance of the radar/sonar system by removing the possible occurrence of spurious targets, as in other codes [39]. A Costas array is inherently a frequency hopping system, and so each bit of the sequence is expected to undergo different attenuation and phase constants when travelling through the same medium. Even though this was not the first design principle of these codes, this fact provides an inherent robustness against the dispersive propagation impairments.

A priori, it could be said that all the codes would offer a similar performance once propagated through the same medium and that their performance would be more conditioned by the scenario rather than by dielectric properties issues. However, we demonstrate in this paper the remarkable differences between the codes that can be observed, such as the peak amplitude decay, the degradation of the related CCF or the multipath detectability. All these three aspects are here taken into account to discuss the differences among codes, and we generally concluded that each case deserves to be studied for the specific aim conditions. 

Throughout the present work, the need to approach the study of compensatory techniques in order to provide mitigation mechanisms for the impairments introduced by the dispersive propagation on the transmitted signals is demonstrated. Traditionally, a mismatched filtering is used to compensate the effect introduced by the medium. However, this technique is not valid in presence of multipath propagation. We then propose an anti-dispersive (AD) filter approach derived in the frequency domain that would help to improve the CCF by restoring the received signal features under multipath conditions. This would be helpful for information retrieval purpose or just to restore the original features of the propagating waveform [40], even in presence of multipath. 

In summary, this article describes a methodology in the frequency domain to analyze the behavior of different codes when propagated through a dispersive medium. There are other analysis solutions that develop close-form solutions in the time domain and are limited to waveforms formed by a pulse [13,14,15,16,17,18,20,21,22,24,27,28,29,30,31,32,33]. The chosen codes were Barker, PRBS, Frank and Costas, as well as WGN. The behavior analysis is carried out in terms of the CCF, analyzing both its deformation due to time broadening, and the degradation of the protection level given by the SSL. The reduction in SSL experienced by the codes can be critical in a scenario where multipath occurs, since secondary lobes could be mistaken for false echoes and vice versa. This requires considering the use of an AD filtering that compensates for the dispersive effect of the channel and also takes into account the presence of the different multipath components (MPC).

In Section 2, firstly, the theoretical analysis of the propagation through a dispersive medium is introduced based on the frequency transfer of the medium. In Section 3, the methodology is described, which was developed to analyze the impairments introduced by the propagation of the random noise waveforms through three different naturally dispersive media—soil, soil and water, vegetation—in terms of peak amplitude decay, CCF degradation, SSL degradation and multipath detectability. The theoretical simulations were performed at a center frequency of 1.5 GHz and for a signal bandwidth of 3 GHz, and the results obtained are commented in Section 4. The last step of the methodological analysis is described in Section 5 with the frequency response for an AD filtering approach. The results achieved for the simulations are also discussed, which were performed considering the signals and dielectric models of this study for a scenario with and without multipath propagation. Finally, a general discussion and conclusions close this paper.

## 2. Theoretical Analysis of Dispersive Propagation

For the purpose of theoretical analysis, a frequency-domain analysis has been assumed as described in [19]. According to this analysis, an input signal *p*(*t*) passes through a medium with a frequency response *H*(*z*,*f*), which would act as a frequency filter or channel response, so that each frequency component *P*(*f_k_*, *z* = 0) is affected differently upon propagation through the dispersive material, modeling the behavior of the different spectral components of the propagating signal *p*(*t*). The final result, *y*(*t,z*), is the dynamical evolution of the signal affected by the medium dispersion at a propagation distance *z*, as indicated in (1):

(1) $$y\left(t,z\right)=F^{-1}\left\{P\left(f,z=0\right)\cdot H\left(f,z\right)\right\}$$

with the $F^{-1}\left\{\cdot \right\}$ indicates the Inverse Fourier Transform. For estimating the medium frequency response or transfer function *H*(*f*,*z*), we considered that the signal impinges from air into an infinite planar half-space medium; then, the response *H*(*f*,*z*) can be seen as a ratio of the electric field transmitted through the medium until a distance *z* within it, and the field *E*_0_ incident in the air–medium interface planar boundary, which becomes the phase and amplitude reference point. Finally, the medium frequency response is given as in (2):

(2) $$H\left(f,z\right)=\frac{E_{medium}^{t}\left(z,t\right)}{E_{0}}=T_{am}\cdot e^{-\gamma_{m}\left(f\right)z}$$

where *T_am_* indicates the transmission coefficient between the air and the medium at the interface; and *γ_m_* is the propagation coefficient of the medium considered given in (1/m), and it is a frequency dependent parameter. We can observe that the medium frequency response *H*(*f*,*z*) is a function of the propagation distance *z*. The expression in (2) is valid if a Fresnel transmission model [41,42] can be assumed, as in the case of large attenuative media or large propagation distances. 

The propagation coefficient *γ_m_* can be derived from the complex relative dielectric permittivity *ε_rm_(f)* of the medium under study, as per (3):

(3) $$\gamma_{m}\left(f\right)=j\frac{2\pi f}{c}\sqrt{\varepsilon_{rm}\left(f\right)}$$

with *c* the speed of light in free space, and *f* the frequency in Hz. 

The large influence of the complex dielectric permittivity is observed on the final result, *y*(*t*,*z*). The dielectric model assumed to characterize the propagation medium becomes critical in order to achieve an accurate propagation analysis. In Section 5, it is explained that the dielectric model also influences the impairment mitigation techniques are designed to achieve the information retrieval or just to restore the received signal.

The dispersive media considered in this paper are the soil, a moisturized version of soil, and vegetation. The complex relative dielectric permittivity for soil *ε_r_*_,*soil*_(*f*) is given as a Rocard-Powles-Debye model [16] as per (4):

(4) $$\varepsilon_{r,soil}\left(f\right)=\sum_{j=1}^{3}\frac{a_{j}}{\left(1-i\cdot \omega \tau_{j}\right)\cdot \left(1-i\cdot \omega \tau_{f}{}_{j}\right)}$$

with constants *a*_1_ = 13.63, *a*_2_ = 0.33, *a*_3_ = 0.293; rotational relaxation times *τ*_1_ = 1.82 × 10^−6^ s; *τ*_2_ = 1.97 × 10^−8^ s; *τ*_3_ = 2.36 × 10^−10^ s; frictional relaxation times *τ_f_*_1_ = 8.78 × 10^−11^ s; *τ_f_*_2_ = 1.12 × 10^−10^ s; *τ_f_*_3_ = 1.00 × 10^−15^ s; and with *ω* the frequency in rad/s. 

For the second kind of soil, a linear mixing model has been fitted using 75% of soil and 25% of triply distilled water (TDW) [25], with an equivalent complex relative dielectric permittivity *ε_r_*_,*soil+TDW*_(*f*) given as in (5):

(5) $$\varepsilon_{r,soil+TDW}\left(\omega \right)=0.75\cdot \varepsilon_{r,soil}\left(\omega \right)+0.25\cdot \varepsilon_{r,TDW}\left(\omega \right)$$

with *ε_r_*_,*soil*_*(f)* derived from (5) and *ε_r_*_,*TDW*_*(f)* given by the Rocard-Powles-Debye model found in [9,10] and with the following general expression (6):

(6) $$\varepsilon_{w}\left(\omega \right)=\varepsilon_{\infty }-\frac{\varepsilon_{S}-\varepsilon_{\infty }}{\left(1-i\cdot \omega \tau \right)\cdot \left(1-i\cdot \omega \tau_{f}\right)}$$

with constants *ε_∞_* = 2.1, *ε_S_* = 76.2, relaxation time *τ*_1_ = 8.44 × 10^−12^ s, frictional relaxation time *τ_f_* = 4.62 × 10^−14^ s for the case of TDW.

For the vegetation *ε_r_*_,*veg*_*(f)*, we considered the Maetzler model [43,44], as in (7):

(7) $$\varepsilon_{r,veg}\left(\omega \right)=0.522\cdot \left(1-1.32\cdot m_{d}\right)\cdot \varepsilon_{sw}\left(\omega \right)+3.84\cdot m_{d}+0.51$$

which is a semi-empirical formula taking into consideration parameters such as the dry-matter fraction *m_d_* and the complex permittivity of saline water *ε_sw_*(*ω*), which basically consists of a Debye model as indicated in (8):

(8) $$\varepsilon_{sw}\left(\omega \right)=\varepsilon_{\infty }-\frac{\varepsilon_{S}-\varepsilon_{\infty }}{\left(1-i\cdot \omega \tau \right)}$$

with constants *ε_∞_* = 5.27, *ε_S_* = 80; and relaxation time *τ*_1_ = 1.00 × 10^−11^ s, according to [43,44,45,46,47]. 

Table 1 summarizes the dielectric models associated with each of the three dispersive media analyzed in this article. Also included is the value of the characteristic parameters of each model.

**Figure 1:**
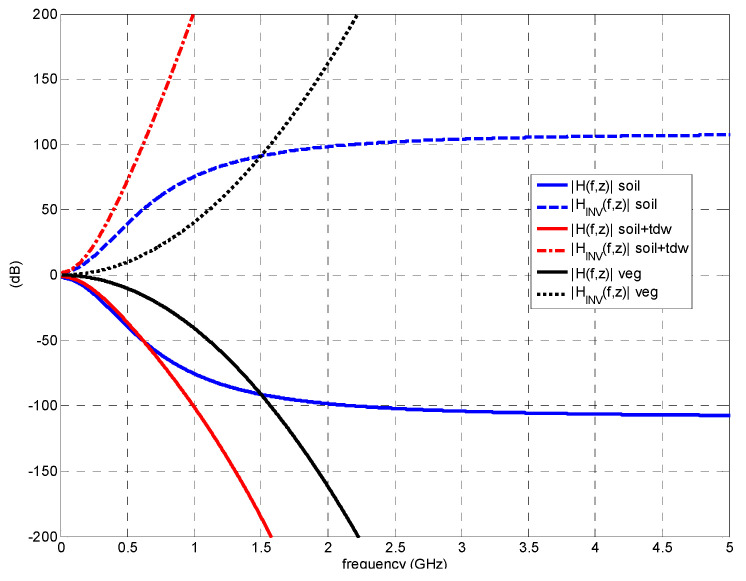
Comparison of frequency responses for the three media *H(f,z)* and the anti-dispersive media *H_INV_(f,z)* according to method of Section 5.

In Figure 1, the response *H(f,z)* for the three media is represented as a function of dielectric value *ε*$\left(\omega \right)$ and, therefore, as a function of frequency, at a given propagation depth *z =* 25∙*z_d_*. A larger attenuative behavior is observed for the moisturized soil and vegetation, whilst the simple soil presents an asymptotic trend for frequencies above 3 GHz. 

**Figure 2:**
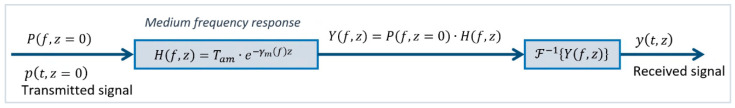
Diagram of the frequency-domain analysis of the propagation through dispersive media.

The theoretical frequency-domain approach described in this Section 2 can be summarized in the diagram shown in Figure 2.

## 3. Methodology

A methodology has been designed in order to analyze the behavior of the different codes regarding the dispersive phenomenon and the multipath presence. This methodology consisted of eleven steps:

1. It was assumed *T_am_* = 1, *f*_0_ = 1.5 GHz and *T_b_* = 1/*f*_0_. Only a linear polarization was considered.
2. Generate the time version $c\left(t\right)$ for a sequence u of each code. The code signal $c\left(t\right)$ represents the baseband information-bearing signal with a chip duration *T_b_*. It is not possible for the four codes (Barker, PRBS, Costas and Frank) to have an identical length M, but the closest one has been chosen.
3. Build the sine modulated transmitted signal $p\left(t,z=0\right)$ as indicated in (9):
  (9) $$p\left(t,z=0\right)=p\left(t\right)=c\left(t\right)\cdot \sin\left(2\pi f_{0}t\right)$$
  with *f*_0_ being the carrier frequency.
4. Calculate the medium frequency response $H\left(f,z\right)$ according to (10):
  (10) $$H\left(f,z\right)=T_{am}\cdot e^{-j\gamma_{m}\left(f\right)z}=e^{-j\frac{2\pi f}{c}\sqrt{\varepsilon_{rm}\left(f\right)}\cdot z}$$
  where the dielectric model $\varepsilon_{rm}\left(f\right)$ and its related parameters are taken from Table 1. The medium is formed by one single layer of infinite thickness, i.e., Fresnel model.
5. Calculate the dynamical evolution of $p\left(t\right)$ as indicated in (11) and (12):
  (11) $$y\left(t,z\right)=F^{-1}\left\{P\left(f,z=0\right)\cdot H\left(f,z\right)\right\}=F^{-1}\left\{P\left(f,z=0\right)\cdot e^{-j\frac{2\pi f}{c}\sqrt{\varepsilon_{rm}\left(f\right)}\cdot z}\right\}$$
  (12) $$y\left(t,z\right)=F^{-1}\left\{P\left(f,z=0\right)\cdot e^{-j\frac{2\pi f}{c}\sqrt{\varepsilon_{rm}\left(f\right)}\cdot z}\right\}=E_{T}\left(z,t\right)$$
  with $z\in \left[0,\;25\cdot z_{d}\right]$ and $z_{d}$ the reference distance given by the penetration distance inside the medium so that $H\left(f,z=z_{d}\right)=e^{-1}$. The received signal $y\left(t,z\right)$ is usually named $E_{T}\left(z,t\right)$ in literature [13,14,15,16,17,18,19,20,21,22,23,24,25,26,27,28,29,30,31,32,33]. The peak amplitude decay as a function of propagation distance is derived from the received signal $E_{T}\left(z,t\right)$.
6. Calculate the CCF between $p\left(t\right)$ and $y\left(t,z\right)$, analyze the degradation of the main peak and side lobes, for each code and propagation medium.

Once the behavior of the different codes is analyzed in terms of dynamical evolution and SSL degradation, the multipath detectability of the codes was studied. To this aim, the following steps were added to the methodology:

7. Generate the input signal as a composite of replicas of the original signal $p\left(t\right)$ obtained in Step 3, so conforming the waveform given in (13) and (14):
  (13) $$p^{\prime }\left(t,z=0\right)=p^{\prime }\left(t\right)=\sum_{k=0}^{3}a_{k}\cdot p\left(t-\tau_{k}\right)=\sum_{k=0}^{3}\frac{4-k}{4}\cdot p\left(t-4\cdot k\cdot T_{b}\right)$$
  (14) $$p^{\prime }\left(t\right)=p\left(t\right)+0.75\cdot p\left(t-4T_{b}\right)+0.5\cdot p\left(t-8T_{b}\right)+0.25\cdot p\left(t-12T_{b}\right)$$
  with $T_{b}$ being the code chip period.
8. Calculate the dynamical evolution of $p^{\prime }\left(t\right)$ as indicated in (15):
  (15) $$y^{\prime }\left(t,z\right)=F^{-1}\left\{P^{\prime }\left(f,z=0\right)\cdot e^{-j\frac{2\pi f}{c}\sqrt{\varepsilon_{rm}\left(f\right)}\cdot z}\right\}=E_{T}^{’}\left(z,t\right)$$
9. Calculate the CCF *X_C_(τ,z)* between $p^{\prime }\left(t\right)$ and $y^{\prime }\left(t,z\right)$, analyze the degradation of the main peak and side lobes, and determine the SSL for each code and propagation medium.
10. Comparison of the CCF degradation in terms of the SSL obtained in Step 9.
11. Compensation of dispersive propagation impairments by means of anti-dispersive filtering. This step is explained in Section 5, for both for the simple case and the case with multipath presence.

**Figure 3:**
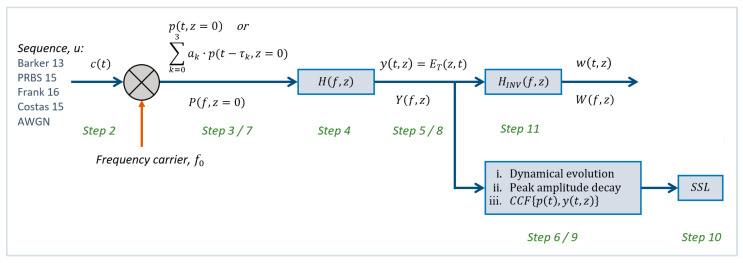
Diagram of the methodology for the analysis of dispersive propagation.

The methodology described in this Section 3 can be summarized in the diagram shown in Figure 3.

The sequences u used for building the base-band signal $c\left(t\right)$ are summarized in Table 2. For the generation of the WGN signal Matlab was used with the parameters also described in Table 2.

For the WGN case, the peak amplitude decay estimated as in Step 3 is meaningless. To obtain more information about the power decay evolution as described in [45], it must be estimated the time-averaged power density $P_{T}^{avg}\left(z\right)$ by means of the averaged Poynting vector. In order to include the dispersive nature of the medium, the *k*th frequency component of the averaged Poynting vector that has travelled a distance *z* inside a given medium, is calculated as indicated in (16):

(16) $${\bar{S}}_{k}^{avg}\left(z\right)=\frac{1}{2}ℜ\left\{{\bar{E}}_{k}\times {\bar{H}}_{k}^{*}\right\}=\hat{z}\frac{1}{2}ℜ\left\{\frac{{\left|{\bar{E}}_{k}\right|}^{2}}{\eta^{*}\left(\omega_{k}\right)}\right\}=\hat{z}\frac{{\left|X\left(f_{k},z=\right)\right|}^{2}}{2\left|\eta \left(\omega_{k}\right)\right|}\cdot e^{-2\alpha_{k}z}\cdot \cos\theta_{\eta_{k}}\;\;\;\left(\frac{W}{m^{2}}\right)$$

where *η_k_* = *η*(*ω_k_*) = *jω_k_μ*/[*α*(*ω_k_*) + *jβ*(*ω_k_*)] = |*η(ω_k_)*|∙exp(*θ_ηk_*) is the intrinsic wave impedance of the medium for the frequency component, *x*(*t*,0) is the amplitude of the *E*-field at *z* = 0 for the *k*th frequency component, and * denotes a complex conjugate operation. Finally, the total time-averaged power $P_{T}^{avg}\;$ at a given distance *z* inside a given dispersive medium is the sum of the power contained by the vector in (16) assuming a unit area. 

Then, only for the WGN case, the Step 3 of the methodology is completed by the estimation of $P_{T}^{avg}\left(z\right)$.

## 4. Results

This section describes the outcomes obtained to evaluate the code performance according to the methodology described in the previous Section 3. The results have been grouped in three main blocks:

i. Dynamical evolution and peak amplitude decay (Section 4.1): results corresponding to the output of the Step 5 of the methodology.
ii. CCF degradation (Section 4.2): results derived from Step 6.
iii. SSL variation and detectability performance in multipath presence (Section 4.3 and Section 4.4): results achieved in Steps 7, 8, 9 and 10.

### 4.1. Dynamical Evolution

**Figure 4:**
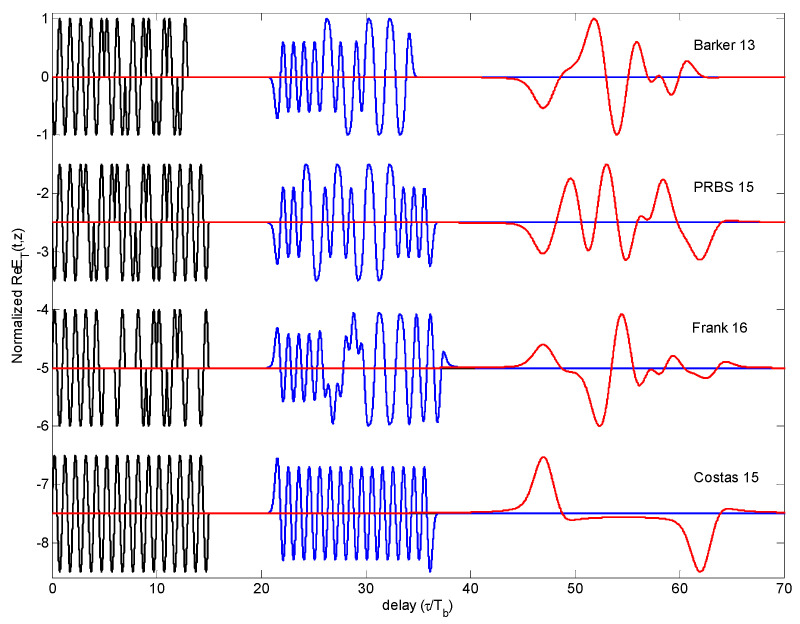
Dynamical evolution of *E_T_*(*z*,*t*) for Barker, PRBS, Frank and Costas codes after propagating through vegetation at input (*z* = 0), *z* = 5∙*z_d_* (second column) and *z* = 15∙*z_d_* (third column).

The dynamical evolution of the received signal $E_{T}(z,t)$ is obtained for each code and the three dispersive media. In Figure 4, the dynamical evolution of the sequences is represented for three propagation distances (0, 5·*z_d_* and 15·*z_d_*) through a single layer of vegetation. 

**Figure 5:**
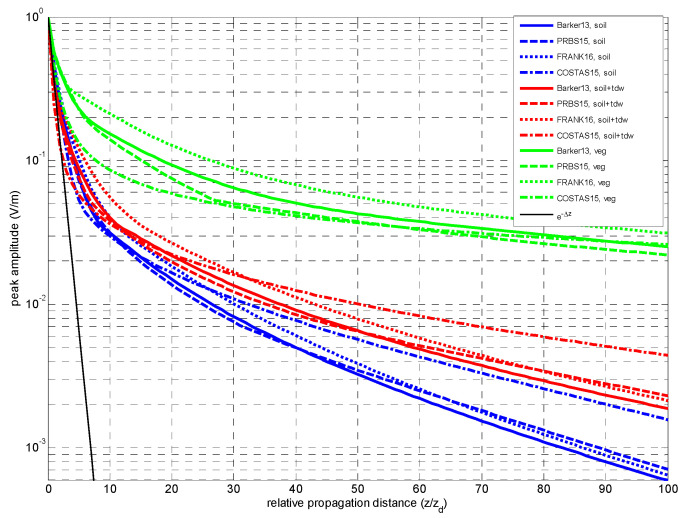
Peak amplitude as a function of propagation distance for three media and binary codes with a chip duration *T_b_* = 1/*f*_0_, at *f*_0_ = 1.5 GHz.

The peak amplitude decay of the received signal $E_{T}\left(z,t\right)$ as a function of propagation distance was represented in Figure 5, comparing the algebraically decay trend obtained for each code and the three dispersive media. Generally, it can be inferred that except for vegetation, the Barker codes seem not to be a good choice in terms of peak decay.

**Figure 6:**
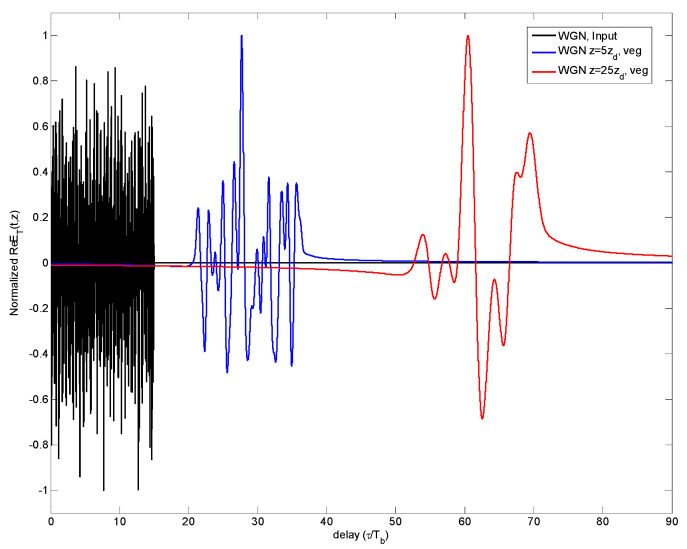
Dynamical evolution of *E_T_*(*z*,*t*) for WGN after propagating through vegetation at input (*z* = 0), *z* = 5∙*z_d_* (second column) and *z* = 15∙*z_d_* (third column).

Even that we have estimated the peak amplitude decay for the WGN, as shown in Figure 5, this result was meaningless, as explained in Section 2. Then, the averaged power $P_{T}^{avg}(z)$ and power extinction factor were estimated. The dynamical evolution of the generated WGN can be followed in Figure 6.

Every code shows a different behavior regarding the dispersive phenomenon. From the Figure 3 to Figure 5 the main conclusions derived are described in the next Section 4.1.1 to Section 4.1.5.

#### 4.1.1. Barker Codes

As shown in the first row of Figure 4, the Brillouin precursor appears as a superimposed wave in the leading and trailing edges of each single pulse; however, as described in [23], the phase transition between adjacent bits reinforces the Brillouin precursor effect by doubling its amplitude level. In the Barker sequence, this reinforcement becomes more evident, as the propagation depth is larger since the precursor becomes the dominant component.

In Figure 5, it is observed that the predominance of the precursor is due to an algebraically decay trend of its peak amplitude level whilst the carrier component undergoes an exponentially decay. This latter trend yields the carrier component faster with propagation distance into the so-called extinction region, in terms of amplitude level.

#### 4.1.2. PRBS Codes

The presence of Brillouin precursor is clear in this sequence, with a dynamical evolution similar to the Barker case, as shown in the second row of Figure 3. However, as observed in Figure 5, the peak decay of the PRBS code indicates a slightly more favorable general performance in comparison with the Barker code.

#### 4.1.3. Frank Codes

If in terms of peak amplitude decay, as observed in Figure 5, it could be concluded that the Frank codes would be the most favorable choice for propagation through any of the chosen media. However, this advantageous performance will not be sustained as later explained in Section 4.2.3.

#### 4.1.4. Costas Codes

A Costas array is a frequency coded sequence, and for this reason each bit experiences a different behavior once travelling inside the medium in terms of attenuation and phase constants. This fact means that the Brillouin precursor corresponding to the trailing edge of one bit is not totally the same as and it is not entirely cancelled by the precursor related to the leading edge of the following bit, due to the absence of phase transition between adjacent bits. 

For the set of selected array and vegetation medium, the remains produced in the intermediate phase transitions are reduced. The result is that the Brillouin precursor is only present in the first leading edge and in the last trailing edge of the sequence, once cancelled the precursors occurred in the intermediate bits. The overall behavior is equivalent to transmit just one rectangular pulse of width duration *M*∙*T_b_*, and so it degrades the multipath resolution capabilities related to the pulse width, as later explained in Section 4.2.4, even though the main use of the Costas arrays is in the Doppler domain. This overall performance consisting of only two observable precursors is shown in Figure 4.

#### 4.1.5. Additive White Gaussian noise

From the power evolution $P_{T}^{avg}\left(z\right)$ shown in Figure 6, the power extinction factor or power attenuation was estimated for the three media and summarized in Table 3. The results indicate an algebraical decay trend followed by the average power of the WGN as a function of the propagation distance *z*. The power attenuation is governed by *b*-10∙*k_e_*∙log_10_(*z*), with *b* = 10∙log_10_(*a*), and the units for the extinction factor *k_e_* are dB/m.

### 4.2. Analysis of CCF Degradation

**Figure 7:**
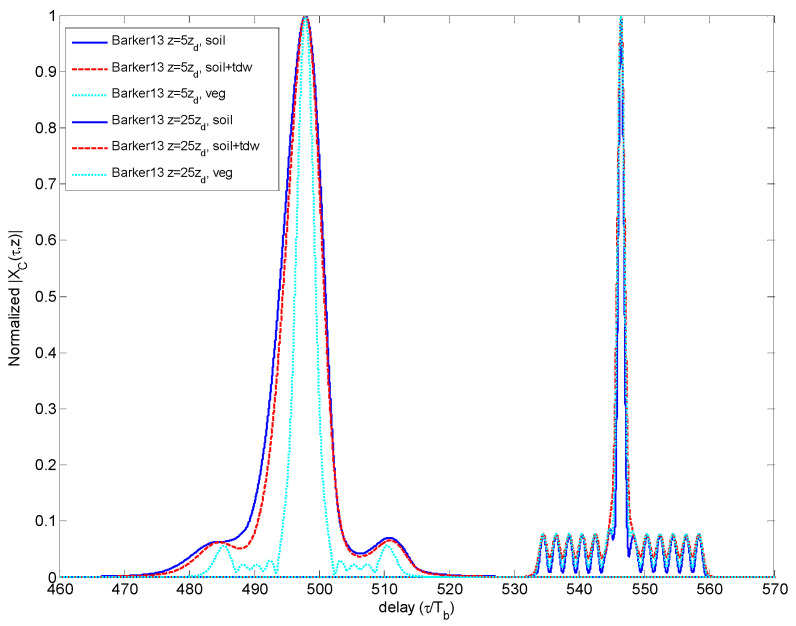
Comparison of CCF for different propagation distances and media for Barker code.

**Figure 8:**
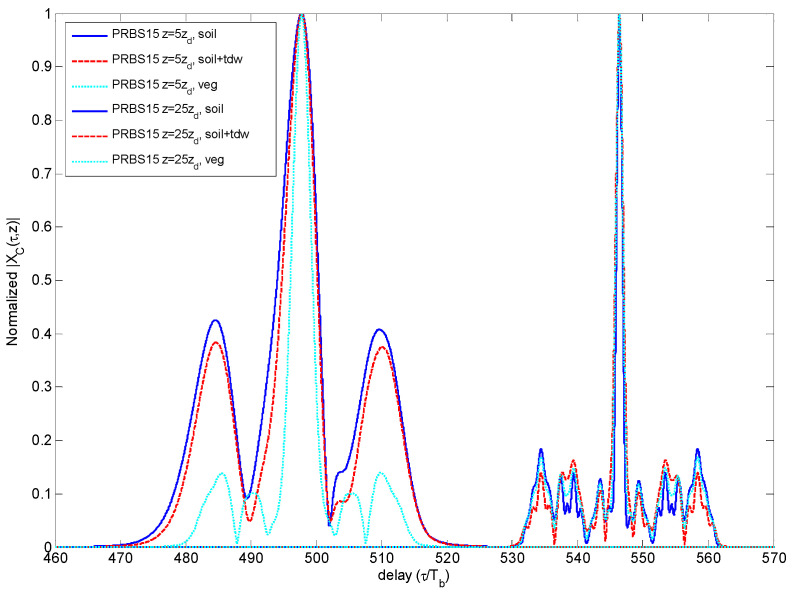
Comparison of CCF for different propagation distances and media for PRBS code.

**Figure 9:**
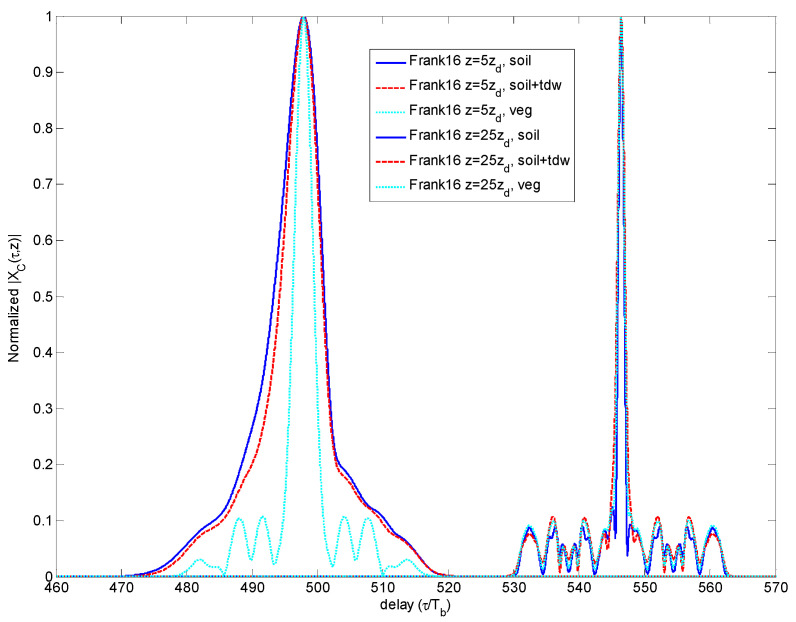
Comparison of CCF for different propagation distances and media for Frank code.

**Figure 10:**
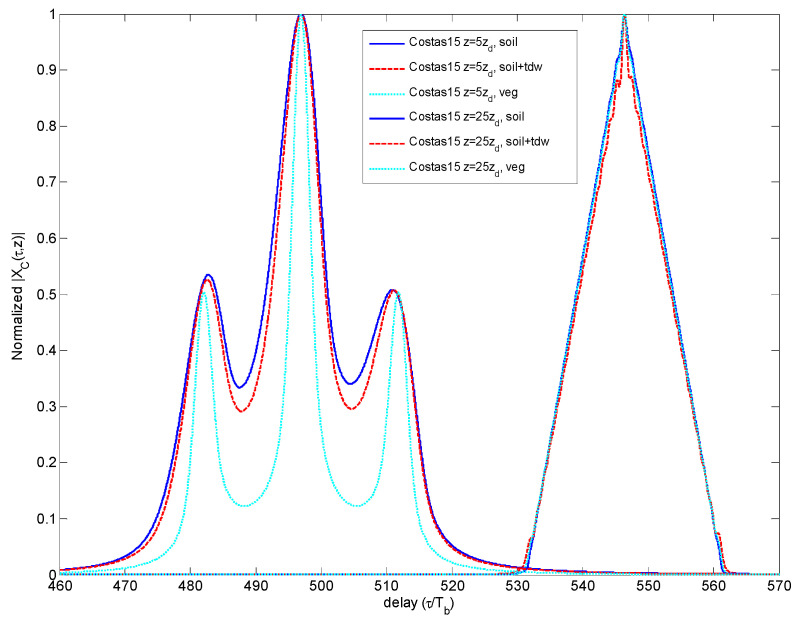
Comparison of CCF for different propagation distances and media for Costas code.

**Figure 11:**
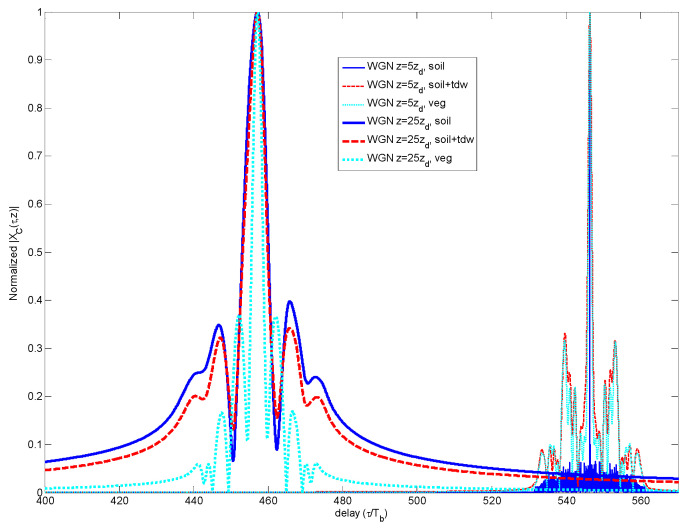
Comparison of CCF for different propagation distances for WGN.

To organize plots and summarize information, Figure 7, Figure 8, Figure 9, Figure 10 and Figure 11 depict the CCF for a short propagation distance (*z* = 5·*z_d_*) on the left side and for a longer distance (*z* = 5·*z_d_*) on the right side, through the three media. 

The deformation evident in the CCF *X_C_(τ,z)* is a consequence of the predominance of the Brillouin component on the propagating signal. Overall, irrespective of the code case, Figure 7, Figure 8, Figure 9, Figure 10 and Figure 11 consistently show CCF degradation with increasing propagation distance, manifested by the descend in the SSL value and the broadening of the CCF peaks/lobes.

With a greater propagation distance within the dispersive medium, there is a proportional increase in time-width broadening and distortion observed in the peaks/lobes of the CCF. Notably, in the right-side CCFs, the time-width broadening leads to the merging of the CCF peaks/lobes. This degradation translates into a loss of resolution in the CCF and, in extreme cases, makes it impossible to detect multipath components.

Although the CCF reference value obtained for *z* = 0 has not been included in Figure 7, Figure 8, Figure 9, Figure 10 and Figure 11, the value represented for *z* = 5·*z_d_* can be considered as a reference, since at this propagation depth the degradation is not critical yet.

The case of each code and the WGN is analyzed in the following subsections.

#### 4.2.1. Barker Codes

Figure 7 compares the CCF obtained for the Barker sequence for two propagation distances (5·*z_d_* and 25·*z_d_*) through each medium. The comparison illustrates that the secondary sidelobes finally merge in just one sidelobe. Nevertheless, the Barker code presents an increase in the SSL of the CCF not as critical as the other codes do.

#### 4.2.2. PRBS Codes

The PRBS sequences have the property of a dynamic range (DR) proportional to the length *M*, given by 10∙log_10_(*M*) (dB). For this reason, a short sequence, as the one used here, present a low DR and, respectively, large SSL given by −10∙log_10_(*M*) (dB). If applied through a dispersive medium, this problem worsens, as observed in the CCF plots of Figure 8. The low DR can limit the PRBS codes performance in terms of both peak amplitude decay and SSL increase.

#### 4.2.3. Frank Codes

If in terms of peak amplitude decay, the Frank codes represented the most favorable choice. However, this evidence is not kept once that the corresponding CCF is analyzed. We observe in Figure 9 the fast broadening occurring to the CCF main peak width. 

#### 4.2.4. Costas Codes

The result is that the Brillouin precursor is only present in the first leading edge and in the last trailing edge of the sequence; once cancelled, the precursors occurred in the intermediate bits. This overall performance consisting of only two observable precursors is shown in Figure 4. It is observed in Figure 10 that the robustness of the Costas code, in terms of CCF degradation, is weak.

#### 4.2.5. Additive White Gaussian Noise

Finally, in Figure 11 we can check the large distortion undergone by the CCF jointly in the secondary sidelobes presence and broadening of the CCF main peak width. 

### 4.3. Multipath Detectability

In Step 7 of the methodology, the waveform used was defined as input signal that simulates the presence of multipath. This signal is a composite of the original signal *p*(*t*, *z* = 0) plus three time delayed versions—*p*(*t – τ*_1_, *z* = 0), *p*(*t – τ*_2_, *z* = 0) and *p*(*t – τ*_3_, *z* = 0)—in order to simulate three echoes with delay positions *τ*_1_ = 4·*T_b_*, *τ*_2_ = 8·*T_b_* and *τ*_3_ = 12·*T_b_*, and with linearly decreasing amplitudes, as indicated in (13).

**Figure 12:**
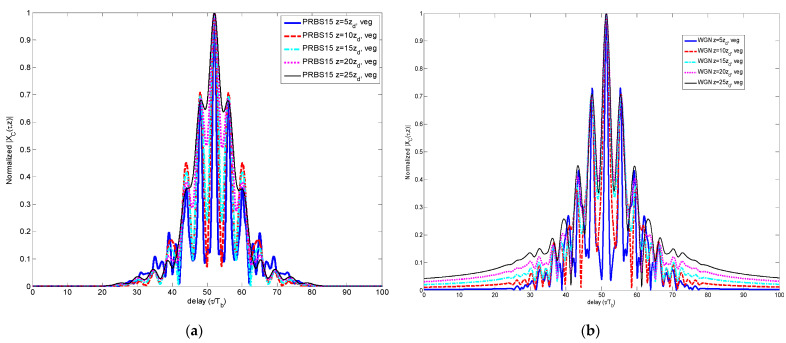
Comparison of CCF for different propagation distances and multipath scenario for (**a**) PRBS and (**b**) WGN.

Once this waveform *p’(t,z =* 0*)* evolves through one of the dispersive media considered here, it becomes *y’(t,z)*, as indicated in Step 8 of the methodology of Section 2. This received signal is correlated with a replica of the original version *p(t,z* = 0*)* in order to detect the multipath presence, thus obtaining the CCF indicated in Step 9. In Figure 12, the CCF is shown for the WGN case, through vegetation as dispersive medium, for different propagation distances *z*.

Provided that the above approach for *p´(t,z* = 0*)* consists of a simplified case, it could be thought of a more complex waveform even with scatterers located inside the medium to achieve a further analysis.

### 4.4. SSL Comparison

In order to quantify the degradation observed in the CCF for the multipath case of the Section 4.3, the SSL values were estimated according to the propagation distance *z* for the different media and codes, according to Step 10 of our methodology. 

**Figure 13:**
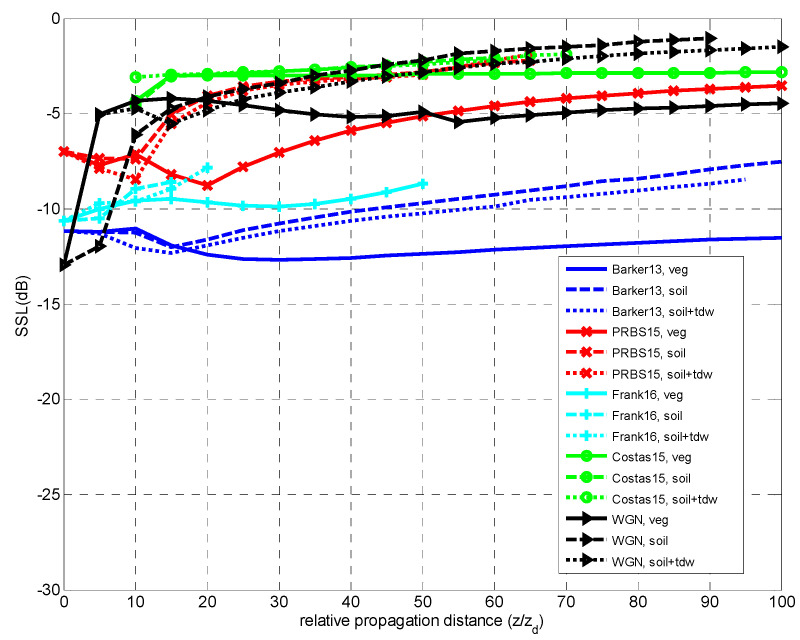
Comparison of SSL vs propagation distance *z* for all of the signals and dielectric media.

The SSL values were derived from the normalized CCF *X_C_(τ,z)* so that it becomes the normalized peak-to-sidelobe (PSL) level. In Figure 13, the SSL estimated for the four codes and WGN is shown in logarithmic units (dB). 

Once more the influence of the underlying medium is observed, and the increase of the SSL values according to the propagation depth *z*. The value at *z* = 0 indicates the SSL value corresponding to the original code, i.e., the ideal value corresponding to an auto-correlation function, and which is not present for the case that it is null, as for the Costas code. 

For all the signal cases, the two types of soils present the less favorable results in terms of SSL values. In particular, the WGN case presents the worst performance. For the Frank code, the broadening of the CCF main peak width masks the secondary peaks, making it impossible to measure the SSL value for longer propagation distances.

## 5. Anti-Dispersive Frequency Filtering

The results presented so far show the need to apply compensatory techniques in order to provide mitigation mechanisms for the impairments introduced by the dispersive propagation on the transmitted signals. This was included in our methodology as Step 11. 

The traditional option consists of designing a filter given by the inverse of the medium frequency transfer as expressed in (17):

(17) $$H_{INV}\left(f,z\right)=\frac{1}{H_{m}\left(f,z\right)}=e^{\gamma_{m}\cdot z}$$

Following the diagram in Figure 3, the signal *w(t,z)* at the output of this filter would be theoretically the same as the original input *p*(*t*,0), as demonstrated in (18):

(18) $$W\left(f,z\right)=Y\left(f,z\right)\cdot H_{INV}\left(f,z\right)=P\left(f,z=0\right)\cdot e^{-\gamma_{m}\cdot z}\cdot e^{\gamma_{m}\cdot z}=P\left(f,0\right)$$

Therefore, the effect is the total cancellation of the dispersion undergone by the input signal *p*(*t*,0). The function *H_INV_*(*f*,*z*) can be seen as the frequency version of a matched filter; however, the matching is carried out with respect to the medium frequency response instead of the transmitted signal. 

In Figure 1, in the solid line plots, the frequency response was shown for the three media—soil, mixing soil and vegetation—for a propagation depth of *z* = 25∙*z_d_*. In dashed line plots, the plots of the inverse filtering *H_INV_*(*f*,*z*) corresponding to the three media are also included. The steep slope of inverse responses indicates how much the AD design accuracy affects the retrieval of the input signal *p*(*t*,0).

Then, in case the dielectric medium is known and thoroughly characterized by a complex relative dielectric permittivity *ε_rm_*(*f*), the information retrieval is possible for any propagation distance travelled by the transmitted signal inside such a medium. Therefore, the impairments produced by the dispersive transmission can be completely mitigated. However, if the dielectric properties of the medium are not exactly known, the dispersive impairments cannot be completely eliminated, and they could even worsen. Thus, the AD filter approach presents some kind of sensitivity or robustness. 

In order to analyze this situation, the effect of the AD filtering on the CCF *X_C_*(*τ,z*) of the waveforms used in this study was analyzed for two cases:

i. Case I: assuming *H_m_*(*f,z*) and *H_INV_*(*f,z*) for the same model of soil as per (6); represents the ideal case.
ii. Case II: with *H_m_*(*f,z*) for soil as in (6) and *H_INV_*(*f,z*) for a model of mixing soil and water given by (7). It represents the situation when the dielectric properties of the medium are not exactly known, and the dispersive impairments cannot be completed eliminated.

**Figure 14:**
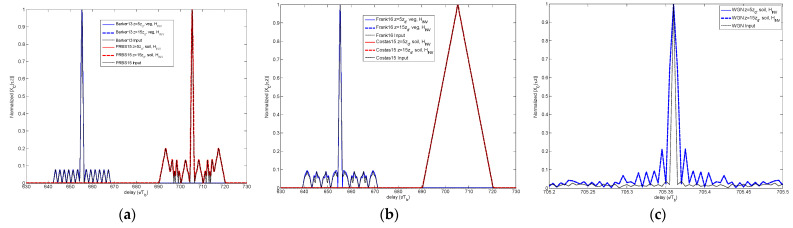
Comparison of CCF obtained in Case I after *H_INV_* filtering for different propagation distances and media for (**a**) Barker and PRBS codes, (**b**) Frank and Costas codes, and (**c**) WGN.

For Case I, as shown in Figure 14a–c, the impairments produced by the dispersive transmission at different propagation distances *z* are completely mitigated and *w*(*t*,*z*) *= p*(*t*,0). It results in a complete inversion of the input signal.

**Figure 15:**
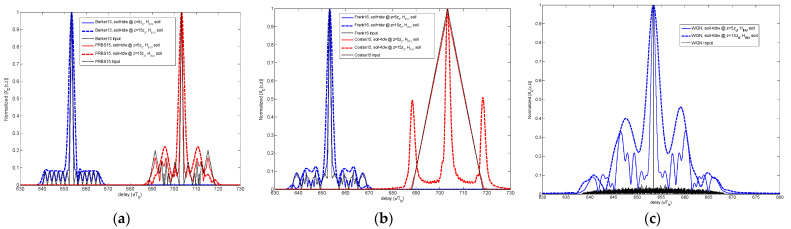
Comparison of CCF obtained in Case II after *H_INV_* filtering for different propagation distances and media for (**a**) Barker and PRBS codes, (**b**) Frank and Costas codes, and (**c**) WGN.

The outputs of Case II are shown in Figure 15a–c: whilst the Barker, PRBS and Frank codes show a relative robustness, the Costas and WGN signals indicate a large sensitivity to the inaccuracy of the inverse filtering that results in a distorted inversion of the input *p*(*t*,0) given that *w*(*t*,*z*) ≠ *p*(*t*,0).

### Multipath Case

Therefore, the inverse filtering inaccuracy is inevitably related, above all, to the knowledge of the propagation medium properties. However, it is also related to the propagation impairments, among which the multipath is undoubtedly a critical phenomenon given its influence on the CCF distortion as demonstrated in Section 4.4.

The existence of MPCs can negatively influence the inverse filtering effectiveness if the AD filter *H_INV_(f,z)* is simply designed as defined in (17), thus degrading the target detection capability of the system. Thus, whether MPC-based information is desired or not, its correct compensation must be taken into account to define an accurate AD filter *H_INV_(f,z)* in a dispersive scenario. 

As an example, it will be considered the following ideal scenario: the direct ray travels through a dispersive medium a distance *z* = *L*, and at that some point the *k*-th MPC of the input signal $p\left(t,z\right)$ coming from a target present within the medium is incorporated. The total distance travelled by the echo till the receiver will be *z* = *X_k_*. At *z* = *L*, the propagated signal $y^{\prime \prime }\left(t,z\right)$ can be described as in (19):

(19) $$y^{\prime \prime }(t,z=L)=p(t,z=L)+B_{k}\cdot e^{j\theta_{k}}\cdot p(t-nT_{b},z=X_{k})$$

The MPC is represented by the term $B_{k}\cdot e^{j\theta_{k}}\cdot p\left(t-nT_{b},z=X_{k}\right)$ where $n_{k}T_{b}$ indicates the excess delay in bin integers; and with $B_{k}\cdot e^{j\theta_{k}}$ being the general complex amplitude value expressed in polar form. For most of the radio channel scenarios, those MPCs amplitudes will be derived from the channel sounding performed with any of the codes used in this paper. It can also be derived from deterministic or quasi-deterministic models [48,49,50,51]. It was ideally assumed that both the direct ray and the echo travel in normal incidence directions and that the target does not introduce any interface needed of characterization by a transmission or reflection coefficient. 

**Figure 16:**
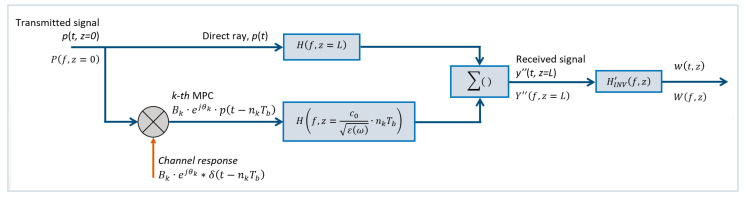
Channel model for the estimation of the AD filtering in presence of multipath.

Therefore, the channel model formed by the direct ray and the MPC can be represented as the parallel of both frequency responses, as illustrated in Figure 16.

Given that the distances travelled by the direct ray and the MPC are different, each term of the received signal will be proportionally affected by the medium frequency response:

○ for the direct ray the medium frequency response will be $H\left(f,z=L\right)=e^{-\gamma_{m}\cdot L}$;
○ for the MPC case, the medium frequency response will be $H\left(f,z=X_{k}\right)$ where $X_{k}$ indicates the distance travelled during a delay time $n_{k}T_{b}$ at a speed of $c_{0}/\sqrt{\varepsilon \left(\omega \right)}$ through a medium of dielectric value $\varepsilon \left(\omega \right)$.

Under this assumption, the direct ray and the multipath component will reach the receiver at *z* = *L* as the signal indicated in (19). This waveform can be expressed in the frequency domain applying (1) leading to (20):

(20) $$Y^{\prime \prime }\left(f,z=L\right)=P\left(f,z=0\right)\cdot H\left(f,z=L\right)+B_{k}\cdot e^{j\theta_{k}}\cdot P\left(f,z=0\right)\cdot e^{-j2\pi f\cdot n_{k}T_{b}}\cdot H\left(f,z=\frac{c_{0}}{\sqrt{\varepsilon \left(\omega \right)}}\cdot n_{k}T_{b}\right)$$

Reordering (20), a simpler expression (21) is obtained:

(21) $$Y^{\prime \prime }\left(f,z=L\right)=P\left(f,z=0\right)\cdot \left[H\left(f,z=L\right)+B_{k}\cdot e^{j\theta_{k}}\cdot e^{-j2\pi f\cdot n_{k}T_{b}}\cdot H\left(f,z=\frac{c_{0}}{\sqrt{\varepsilon \left(\omega \right)}}\cdot n_{k}T_{b}\right)\right]$$

Once this waveform passes through the anti-dispersive filter *H_INV_*(*f,z = L*), defined in (17), assumed as ideally matched to the underlying medium represented by $H\left(f,z\right)=e^{-\gamma_{m}\cdot z}$, the output is described per (22)–(25):

(22) $$W\left(f,z=L\right)=Y^{\prime \prime }\left(f,z=L\right)\cdot H_{INV}\left(f,z=L\right)=Y^{\prime \prime }\left(f,z=L\right)\cdot e^{\gamma_{m}\cdot \left(z=L\right)}$$

(23) $$W\left(f,z=L\right)=P\left(f,z=0\right)\cdot \left[e^{-\gamma_{m}\cdot L}+B_{k}\cdot e^{-j\left(2\pi f\cdot nT_{b}-\theta_{k}\right)}\;\cdot e^{-\gamma_{m}\cdot \frac{c_{0}}{\sqrt{\varepsilon \left(\omega \right)}}\cdot nT_{b}}\right]\cdot e^{\gamma_{m}\cdot L}$$

(24) $$W\left(f,z=L\right)=P\left(f,z=0\right)\cdot \left[1+B_{k}\cdot e^{-j\left(2\pi f\cdot nT_{b}-\theta_{k}\right)}\cdot e^{-\gamma_{m}\cdot \left(\frac{c_{0}}{\sqrt{\varepsilon \left(\omega \right)}}\cdot nT_{b}-L\right)}\;\right]$$

(25) $$W\left(f,z=L\right)=P\left(f,z=0\right)+P_{MPC}\left(f,z\right)$$

From (25), it can be inferred that the multipath component $P_{MPC}\left(f,z\right)$ cannot be totally compensated by the classical inverse filtering as described in (17). Only the direct ray contribution can be inverted by the classical AD filtering. From the received signal it would be required to determine the echo delay positions occurring at *n*·*T_b_* and its amplitudes to achieve a total compensation of the multipath component $P_{MPC}\left(f,z\right)$. Once this term is correctly compensated, it can be eliminated or used for target detection [52,53,54] or at least for minimizing its effect on the desired signal $P\left(f,z=0\right)$. This simple case is an illustrative example of the complexity related to the named inverse problem in radar imaging.

Then, finally, the expression for the AD filtering under multipath conditions $H_{INV}^{’}\left(f,z\right)$ would be given by (26) for the case of an arbitrary number of MPCs or multipath clutter:

(26) $$H_{INV}^{’}\left(f,z\right)=\frac{1}{e^{-\gamma_{m}\cdot z}+\sum_{k=1}^{N}\left(B_{k}\cdot e^{-j\left(2\pi f\cdot nT_{b}-\theta_{k}\right)}\;\cdot e^{-\gamma_{m}\cdot X_{k}}\right)}$$

where *n_k_* denotes the excess delay related to the *k*-th MPC and *X_k_* is the distance travelled by this echo, which in turn depends on *n_k_*:

(27) $$X_{k}=\frac{c_{0}}{\sqrt{\varepsilon \left(\omega \right)}}\cdot n_{k}T_{b}$$

The expression in (26) is the frequency response of the generalization of *N* + 1 parallel systems as depicted in Figure 16, where the first system corresponds to the direct ray and the other *N* parallel branches would correspond to each of the MPCs.

## 6. Discussion

In the present paper the effects of the dispersive propagation have been extensively analyzed on four binary like-noise sequences and one analog random noise signal. The theoretical analysis was carried out in the frequency domain considering the underlying medium as a frequency filter. An eleven-step methodology has been designed to define different analysis objectives of the chosen waveforms, even in the presence of multipath, regarding the dispersive phenomenon: dynamical evolution, peak amplitude decay or power extinction factor, CCF degradation, SSL evolution, and dispersive compensation. 

The analysis has considered the Brillouin precursor formation as the main reason to explain the impairments observed in the signal once propagated through three naturally dispersive media: soil, moisturized soil and vegetation. For deep propagation distances within these media, the precursor becomes the dominant component with related amplitude several orders of magnitude larger than the level corresponding to the frequency carrier component. This fact leads to distortions of the received sequences, in shape and chip duration, that become critical for the proper estimation of the CCF. 

The Brillouin precursor appears as a super imposed signal on the leading and trailing edges of each pulse of the binary sequences. For phased codes, such as Barker, PRBS and Frank, the bit phase transitions produce a cancellation or two-folding of the precursor amplitude, in a total or partial form. However, for a frequency coded Costas array, each bit or pulse hopes within a different frequency interval and so the sequence is differently influenced by the medium in terms of attenuation and phase constants. The result is that only the Brillouin precursors corresponding to the first and last bit of the sequence are visible.

In this paper we relate the observed CCF degradation to the Brillouin precursor formation, and not to a bandwidth loss as in [40], to become an innovative analysis. For all the signals and media herein studied, two main impairments were observed in the CCF: the broadening of the main peak width and the increase of the SSL. A simple scenario with coherent targets was analyzed to check the performance of the different signals. The results indicate that the WGN case achieves a better performance whilst the binary codes undergo a broadening of the CCF main peak and target related peaks so that the echo detection becomes impossible after a given propagation distance within the medium. 

The results show that the impairments introduced by the dispersive propagation can be mitigated by using filters, which take into account the propagation constant of the underlying medium but also the multipath elements occurring. The AD filter given by (26) considering the multipath clutter would require it to be implemented in an active option by digital processing; however, the AD filter given in (17) could be implemented in a passive structure, by a material with a specific frequency response. The filter proposed in (26) becomes largely suitable for receiver structures implementing a cross-correlation based processing. 

From all these aspects here taken into account to discuss the differences found among the performance of the chosen codes, it can be generally concluded that each case of medium and waveform deserves to be studied for the specific conditions of interest. The analysis methodology here described provides a general scope tool that allows for studying more aspects of dispersive propagation than those considered here. For instance, it may result in an interest for a correct design of the transmitted signal [25,46], or for a correct MPC removal [52,53].

## 7. Conclusions

In this paper, the methodological analysis of the Brillouin precursor formation was reported, arisen due to the propagation of wide and ultra-wide band signals in trough frequency dispersive media and show their role in the multipath detection for applications such as radar. This is significant because the impairments introduced by the Brillouin precursor formation cannot be counteracted by a simple inverting filter. It was demonstrated here that multipath clutter must be taken into account to adequately compensate the received signal, but that it must also be done under the perspective of the frequency dispersion effect. Finally, an approach to a filter that inverts the dispersive effects even with multipath clutter was proposed. Although the frequency dispersion effect is well-known, and published papers analyzing its importance exist, it is still not considered common to include the analysis of its effect in applications that involve dispersive media, such as soil, water, sand, vegetation or the ionosphere. In this paper, a further step in this research field has been taken to illustrate that this effect, together with the presence of a clutter of multipath components that have also experienced frequency dispersion, is even more noticeable. With the proposed analysis methodology and the achieved results, it is expected that forthcoming research will take these conclusions into consideration to accomplish, for example, improvements in radar imaging, bathymetry or seabed soundings.

## Figures and Tables

**Table 1:** Dielectric model for the three media considered.

Medium	Dielectric Model	Parameters
Soil	$\varepsilon_{r,soil}\left(f\right)=\sum_{j=1}^{3}\frac{a_{j}}{\left(1-i\cdot \omega \tau_{j}\right)\cdot \left(1-i\cdot \omega \tau_{f}{}_{j}\right)}$	*a*_1_ = 13.63, *a*_2_ = 0.33, *a*_3_ = 0.293 *τ*_1_ = 1.82 × 10^−6^ s; *τ*_2_ = 1.97 × 10^−8^ s; *τ*_3_ = 2.36 × 10^−10^ s *τ_f_1__* = 8.78 × 10^−11^ s;* τ_f_2__* = 1.12 × 10^−10^ s; *τ_f_*_3_ = 1.00 × 10^−15^ s
Mixing soil	$\varepsilon_{r,soil+TDW}\left(\omega \right)=0.75\cdot \varepsilon_{r,soil}\left(\omega \right)+0.25\cdot \varepsilon_{r,TDW}\left(\omega \right)$	*ε_∞_* = 2.1, *ε_S_* = 76.2
	$\varepsilon_{TDW}\left(\omega \right)=\varepsilon_{\infty }-\frac{\varepsilon_{S}-\varepsilon_{\infty }}{\left(1-i\cdot \omega \tau \right)\cdot \left(1-i\cdot \omega \tau_{f}\right)}$	*τ_f_* = 4.62 × 10^−14^ s
Vegetation	$\varepsilon_{r,veg}\left(\omega \right)=0.522\cdot \left(1-1.32\cdot m_{d}\right)\cdot \varepsilon_{sw}\left(\omega \right)+3.84\cdot m_{d}+0.51$	*ε_∞_*= 5.27, *ε_S_* = 80
	$\varepsilon_{sw}\left(\omega \right)=\varepsilon_{\infty }-\frac{\varepsilon_{S}-\varepsilon_{\infty }}{\left(1-i\cdot \omega \tau \right)}$	*τ*_1_ = 1.00 × 10^−11^ s

**Table 2:** Sequences *u* generated for each code.

Code	*Length,* M	Generated Sequence, *u*
Barker	13	[−1,−1,−1,−1,−1,1,1,−1,−1,1,−1,1,−1]
PRBS	15	[−1,−1,−1,1,−1,−1,1,1,−1,1,−1,1,1,1,1]
Frank	16	[1,1,1,1,1,1i,−1,−1i,1,−1,1,−1,1,−1i,−1,1i]
Costas	15	*u* = {exp(*j*2*πf_n_t*)}, with *t*∈[0, *T_b_*], *f_n_* = *a_n_*/*T_b_*, *a_n_* = [1,7,10,15,13,14,9,2,6,8,4,3,11,5,12]
WGN	---	Bandwidth = 1/*f*_0_, power = +3 dBm, load 50 Ω

**Table 3:** Extinction Factor *ke* for WGN case.

Medium	*a* [dB]	*ke* [dB/m]
Soil	−82	0.1085
Mixing soil	−87	0.0855
Vegetation	−92	0.0485

## Data Availability

Data is unavailable due to privacy.
